# Performing ICSI within 4 hours after denudation optimizes clinical outcomes in ICSI cycles

**DOI:** 10.1186/s12958-020-00587-y

**Published:** 2020-04-14

**Authors:** Yini Zhang, Yongzhuang Ma, Zishui Fang, Shiqiao Hu, Zhou Li, Lixia Zhu, Lei Jin

**Affiliations:** 1 Reproductive Medicine Center, Tongji Hospital, Tongji Medicine College, Huazhong University of Science and Technology, 1095 JieFang Avenue, Wuhan, 430030 People’s Republic of China; 2 Department of Orthopedics, Tongji Hospital, Tongji Medical College, Huazhong University of Science and Technology, Wuhan, Hubei People’s Republic of China

**Keywords:** Clinical pregnancy rate, Denudation, Fertilization rate, ICSI, Interval

## Abstract

**Background:**

The study aimed to investigate whether and how general and partial time intervals between processes, from human chorionic gonadotrophin (HCG) trigger to intracytoplasmic sperm injection (ICSI), affected the laboratory and reproductive outcomes in ICSI cycles.

**Methods:**

This was a retrospective data analysis of 3602 women who underwent ICSI treatment cycles using partner or donor sperms, performed at Reproduction Medicine Center of Tongji Hospital of Tongji Medical College of Huazhong University of Science and Technology (Wuhan, China) between October 2016 and September 2018. The clinical pregnancy rate was the major outcome in the study. The fertilization and available embryo rates were secondary outcomes.

**Results:**

Data from 3602 consecutive fresh ICSI cycles was analysed. Multivariate linear regression and logistic regression analysis of factors related to fertilization and clinical pregnancy rates showed that fertilization rate (*P* = 0.001) and clinical pregnancy rate (*P* = 0.037) were significantly associated with denudation (DN)-ICSI interval. Long DN-ICSI interval was associated with higher rate of fertilization than short DN-ICSI interval but significantly decreased clinical pregnancy rate when the interval is over 4 h (*P* < 0.05).

**Conclusions:**

DN-ICSI time interval can act as an independent predictor for clinical outcomes in ICSI cycles. The optimal time for ICSI is within 4 h after oocyte denudation for excellent laboratory and reproductive outcomes in ICSI cycles.

## Background

Intracytoplasmic sperm injection (ICSI) was born to primarily solve the infertility problems due to male factors and failure in vitro fertilization (IVF) treatments. In order to get more successful ICSI outcomes, the operation and its optimal timings including oocyte pick up (OPU), denudation (DN) and ICSI in ICSI procedures have been investigated [1, 2].

The viewpoint finds general acceptance that DN and ICSI should be performed for a while after OPU in order to get sufficient time for achieving the maturation procedure [1]. Because oocytes need some time to get complete maturity so that oocytes can be made full use of the somatic cell compartment. While, the reported optimal timings for DN and ICSI could be various between reproductive medicine centers and among patients [2].

Satoshi Mizuno et al. [1] showed the presence of intact cumulus cells during the preincubation period for ICSI should be considered as a critical factor in fertilization and embryonic development, therefore, oocytes needed to incubate some time before denudation. But recently a series of studies about the murine cumulus cell-oocyte complex (COC) indicated that prolonging the oocyte culture with intact cumulus cells could induce apoptosis. That was why DN might be carried out as quickly as possible after oocyte pick up [3–5].

The traditional view is that the denudation operation may damage the cell membrane of the oocyte, which may be caused by the chemical damage of the hyaluronidase and the mechanical damage of the pipette. Because oocytes should recover for a period of time after denudation [2], it is reasonable to conduct ICSI for a while after denudation. Furthermore, incubation of oocytes before ICSI may induce cytoplasmic maturation, allowing oocytes to gain full activation and normal development potential, which may increase fertilization rate and pregnancy rate.

However, Catherme patrat et al. [6] reported the time between denudation and ICSI was negatively correlated with fertilization rate. With the prolongation of time interval between denudation and ICSI, the fertilization rate gradually decreased. It was recommended that sperm injection should be performed immediately after denudation.

Though so many researches have tried to confirm the optimal time for OPU, DN and ICSI in ICSI procedures [7–12], no consistent agreement has been achieved.

In order to obtain an optimal clinical outcome in ICSI cycles, we investigated the large cohort of successive ICSI instances to explore whether and how general and partial time intervals between processes, from human chorionic gonadotrophin (HCG) trigger to sperm microinjection recorded by the operator specifically, affected the laboratory and clinical outcomes in assisted reproductive technology (ART) treatments.

## Methods

### Study population and ethical approval

In the present retrospective study, data were reviewed from women who underwent ICSI treatment cycles using partner or donor sperms, performed at Reproduction Medicine Center in the Tongji Hospital between October 2016 and September 2018. Each of the patients signed written informed consent. This study conformed to the Declaration of Helsinki for Medical Research involving Human Subjects. Meanwhile, Institutional Review Board approval was acquired from the Ethical Committee. All patients had signed authorization to receive their clinical data for analysis later at the beginning of ICSI treatment cycles. Besides,in order to avoid bias in the analysis, we set the exclusion criteria as follows: (a) the patients who had few oocytes retrieved(≤4); (b) the patients who had low fertilization rate(≤25%); (c) women or partner with chromosomal abnormality; (d) the patients who used frozen–thawed oocytes or donor oocytes for fertilization; (e) the patients whose purpose were preimplantation genetic testing (PGT); (f) males with obvious sperm morphology abnormality; (g) sperms from either testicular sperm aspiration or microsurgical epididymal sperm aspiration.

### Protocol for controlled ovarian hyperstimulation (COH)

All patients in our research underwent controlled ovarian hyperstimulation (COH). Ovarian stimulation was carried out using follicle-stimulating hormone (FSH) (Gonal-F, EMD Serono, Rockland, MA, USA) combined with gonadotropin-releasing hormone (GnRH) agonist (Decapeptyl; Ferring, Kiel, Germany) and antagonist (Cetrotide; Merck Serono, Darmstadt, Germany). Even, the methods of ovarian stimulation protocols in our reproduction medicine center contains GnRH agonist long protocol, GnRH agonist short protocol, GnRH antagonist protocol and luteal phase ovarian stimulation, as described elsewhere [13]. The ovarian stimulation protocols and the daily dose of FSH injection were performed according to female ages, ovarian reserve and various reaction to ovarian stimulation in previous cycles. Follicular developments were monitored with ultrasound scanning. Then 250 mg of recombinant Human Chorionic Gonadotropin (hCG) (Ovidrel; Merck Serono, Darmstadt, Germany) was used to for ovulation triggering when three leading follicles reached a mean diameter of 17 mm or two leading follicles reached a mean diameter of 18 mm. Normally, performing oocytes retrieval transvaginally was at least 36 h after HCG trigger. Additionally, embryos were transferred on Day3 and all the transferred embryos were at least six cells with even blastomeres and < 20% fragmentation.

### Timing control

**Fig. 1:**
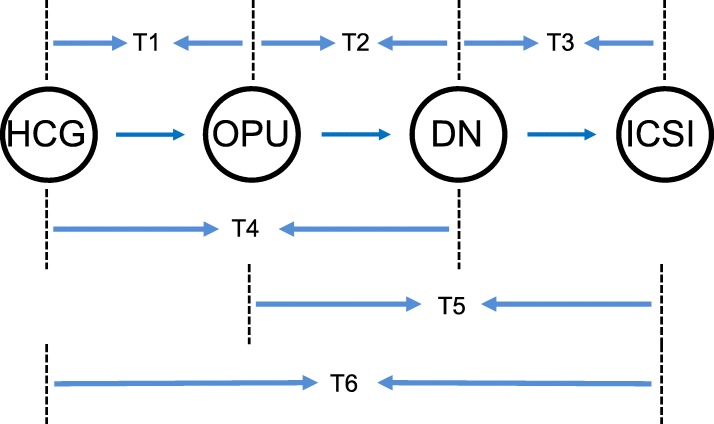
General and partial time intervals between processes, from HCG trigger to ICSI

Exact times including HCG, OPU, DN and ICSI were recorded by the operator to establish a databank of exact times. The detailed times collected were analysed to evaluate the relationship of general and partial time intervals between processes, from HCG to sperm microinjection, and ICSI outcomes including the fertilization rate, available embryo rate and clinical pregnancy rate. In particular, we identified the time interval between HCG trigger and oocyte pick up as T1, oocyte pick up and denudation interval as T2, denudation and ICSI interval as T3, HCG trigger and denudation as T4, oocyte pick up and ICSI interval as T5 and HCG trigger and ICSI interval as T6 (Fig. 1).

### Outcome measure

We regarded clinical pregnancy rate as the major outcome in our research. While, the fertilization and available embryo rates were secondary outcomes. Clinical pregnancy was defined as the presence of a gestational sac with fetal heartbeat 4 weeks after transfer.

### Statistics

SPSS 25.0 (IBM, Armonk, NY, USA) was used to analyze the study data and GraphPad Prism version 6 (GraphPad Software, La Jolla, CA, USA) was used for graph preparation. Moreover, correlation analysis, multivariate linear regression analysis and logistic regression analysis were used to study the effect of times on laboratory and reproduction rates in ICSI cycles. The interval between DN and ICSI varied between 0 min and 340 min (mean 166.51 ± 60.29 min). To rule out bias in the results by assuming that any relationship between DN-ICSI interval and the clinical pregnancy rate may be linear, patients were divided into five distinct groups according to the DN-ICSI intervals: 0–1.00,1.00–2.00, 2.00–3.00, 3.00–4.00, 4.00–5.00 and > 5.00 h for further analysis and confirmation. Equal intervals were set according to these cut-off points for exploring the threshold values. Additionally, associations between demographic and clinical characteristics of the patients were assessed with the use of Student t test or Mann-Whitney U test for continuous variables and chi-square for categoric variables, as appropriate. Results are expressed as mean ± SD. *P* value less than 0.05 was statistical significance for all data analysed.

## Results

The study included a total of 3602 fresh ICSI cycles between October 2016 and December 2018. Table 1 demonstrates the demographic characteristics, cycle characteristics and reproductive outcomes of included cases. The mean age of women in the study was 30.76 ± 4.73 years (range 20–49 years). The mean BMI of women in the study was 21.91 ± 4.68 kg/m2. Percentage of primary infertility is 72.3%. The mean duration of infertility was 3.79 ± 2.84 years. The mean basal FSH was 7.50 ± 2.21 IU/L. The mean number of oocytes retrieved was 13.36 ± 6.23. The fertilization and clinical pregnancy rates were 75.59 and 55.31%, respectively.

**Table 1:** Demographic characteristics, cycle characteristics, and reproductive outcomes of included cycles

Parameter	Value
No. of cycles	3602
Age (y)	30.76 ± 4.73
BMI (kg/m^2^)	21.91 ± 4.68
Percentage of primary infertility (%)	72.3
Duration of infertility (y)	3.79 ± 2.84
Basal FSH (IU/L)	7.50 ± 2.21
No. of oocytes retrieved	13.36 ± 6.23
Fertilization rate (%)	75.59 ± 17.85
Clinical pregnancy rate (%)	55.31
HCG-OPU interval (min)	2218.34 ± 27.00
OPU-DN interval (min)	130.60 ± 46.53
DN-ICSI interval (min)	166.51 ± 60.29

Note: Data are presented as mean ± SD or n (%)

*BMI* body mass index, *OPU* oocyte pick-up, *DN* denudation, *ICSI* intracytoplasmic sperm injection

Correlation analysis of T1-T6 associated with reproduction rates (Table 2) showed the T3, T5 and T6 were significantly correlated with the fertilization rate and T3 was significantly correlated with the clinical pregnancy rate. By contrast, there was no significant correlation of T1, T2 and T4 with: the fertilization or clinical pregnancy rates.

**Table 2:** Correlation analysis of factors related to the fertilization rate and clinical pregnancy rate in ICSI cycles

Time interval	Fertilization rate		Clinical pregnancy rate	
	Pearson C.C.	*P* value	Pearson C.C.	*P* value
T1	0.013	0.439	0.023	0.279
T2	−0.018	0.285	0.009	0.650
T3	0.105	< 0.001^a^	−0.042	0.042^b^
T4	−0.013	0.430	0.028	0.174
T5	0.074	< 0.001^a^	−0.029	0.169
T6	0.088	< 0.001^a^	−0.023	0.267

*Pearson C.C*. Pearson correlation coefficients

^a^Correlation is significant < 0.001 level

^b^Correlation is significant < 0.05 level

The multivariate linear regression and logistic regression analysis of factors related to the fertilization and clinical pregnancy rates is shown in Table 3. The fertilization rate (*P* = 0.001) and clinical pregnancy rate (*P* = 0.037) were significantly related to DN-ICSI interval in ICSI cycles.

**Table 3:** Multivariate linear analysis and logistic regression analysis of factors related to the fertilization rate and clinical pregnancy rate in ICSI cycles

Time interval	*P* value	
	Fertilization rate	Clinical pregnancy rate
T3	0.001	0.037
T5	0.219	0.281
T6	0.314	0.130

**Fig. 2:**
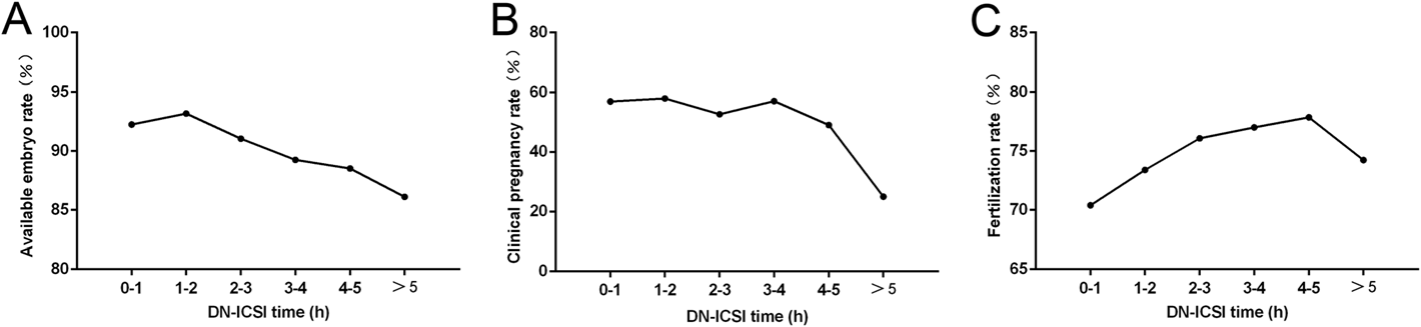
The overall association between the DN-ICSI interval and fertilization rate, available embryo rate and clinical pregnancy rate

Figure 2 shows the overall association between DN-ICSI interval and the fertilization, available embryo and clinical pregnancy rates. In order to get detailed correlation, DN-ICSI time interval was divided into different parts: 0–1.00, 1.00–2.00, 2.00–3.00, 3.00–4.00, 4.00–5.00 and > 5.00 h. Obviously, there was a raise in the fertilization rate with longer DN-ICSI interval within 5 h and decline with progressively prolong DN-ICSI interval after 5 h. In addition, the available embryo rate was higher within 0–2 h post DN and decreased thereafter. With an increase of time between DN and ICSI, clinical pregnancy rate decreased correspondingly progressively in ICSI cycles.

**Fig. 3:**
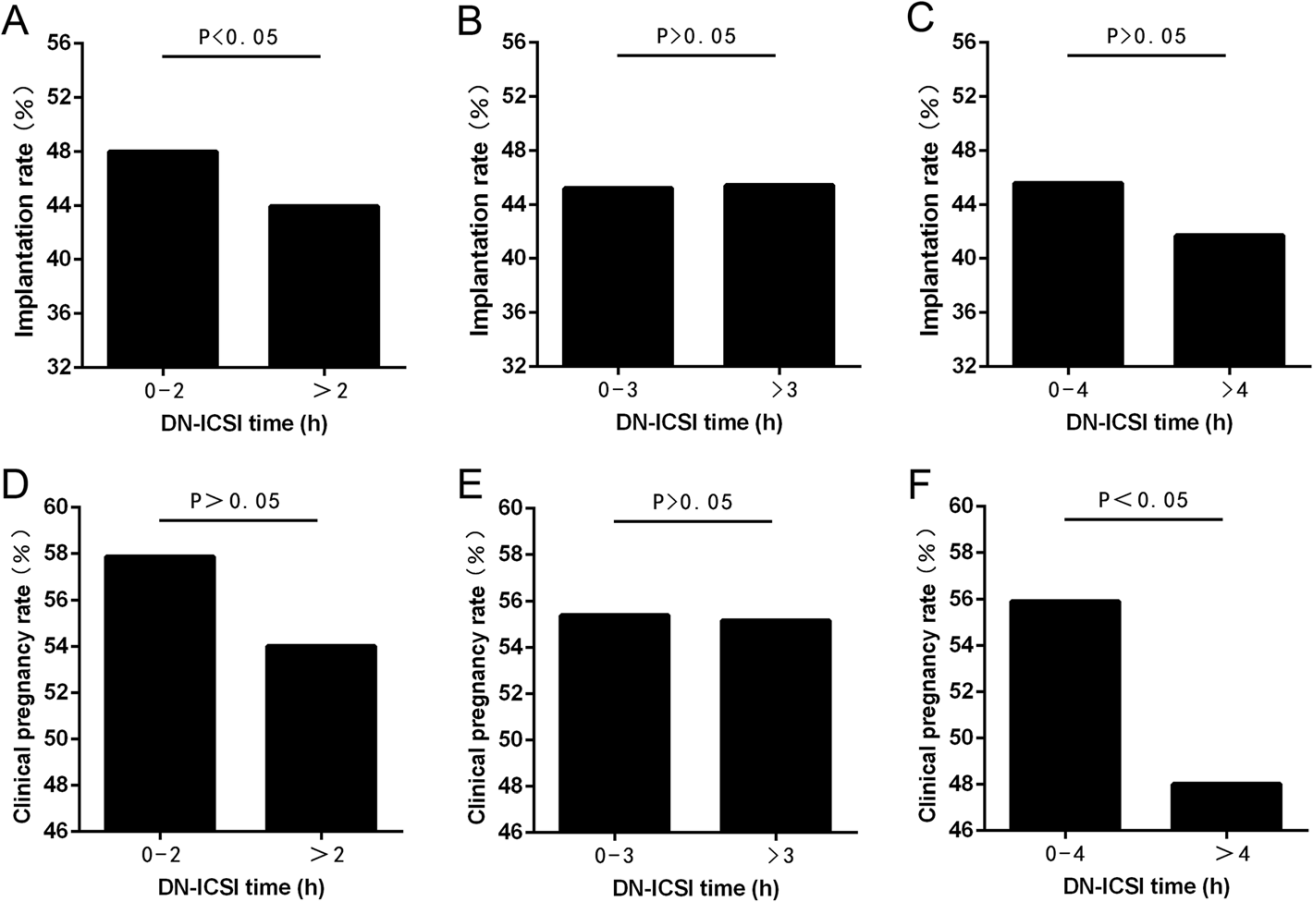
Implantation rate differs according to DN-ICSI time cut-off points. **a** DN-ICSI time cut-off point,2 h; **b** DN-ICSI time cut-off point,3 h; **c** DN-ICSI time cut-off point,4 h. Clinical pregnancy rate differs according to DN-ICSI time cut-off points. **d** DN-ICSI time cut-off point,2 h; **e** DN-ICSI time cut-off point,3 h;(F)DN-ICSI time cut-off point,4 h

Furthermore, when DN-ICSI interval was within 4–5 h, the clinical pregnancy rate was significantly lower (*P* < 0.05) than DN-ICSI interval < 1.0 h, also in DN-ICSI interval > 5 h. Figure 2 also outlines there was an obvious decline of clinical pregnancy rate when DN-ICSI interval was > 4 h. Therefore, in order to find an appropriate cut-off point, DN-ICSI interval of 2 h, 3 h and 4 h were referred as cut-off points to confirm the consequences. Meanwhile, for avoiding the effect of the number of embryos transferred on the clinical pregnancy rate, we specially calculated the implantation rate between groups as assigned and found that there was no difference in the implantation rate of later two groups except the group in which cut-off point was 2 h (Fig. 3). Figure 3 demonstrates that clinical pregnancy rate was significantly different (*P* < 0.05) in DN-ICSI interval < 4 h and > 4 h. Thus, clinical pregnancy rate was significantly higher (*P* < 0.05) in DN-ICSI interval < 4 h compared to DN-ICSI interval > 4 h with similar implantation rates.

**Fig. 4:**
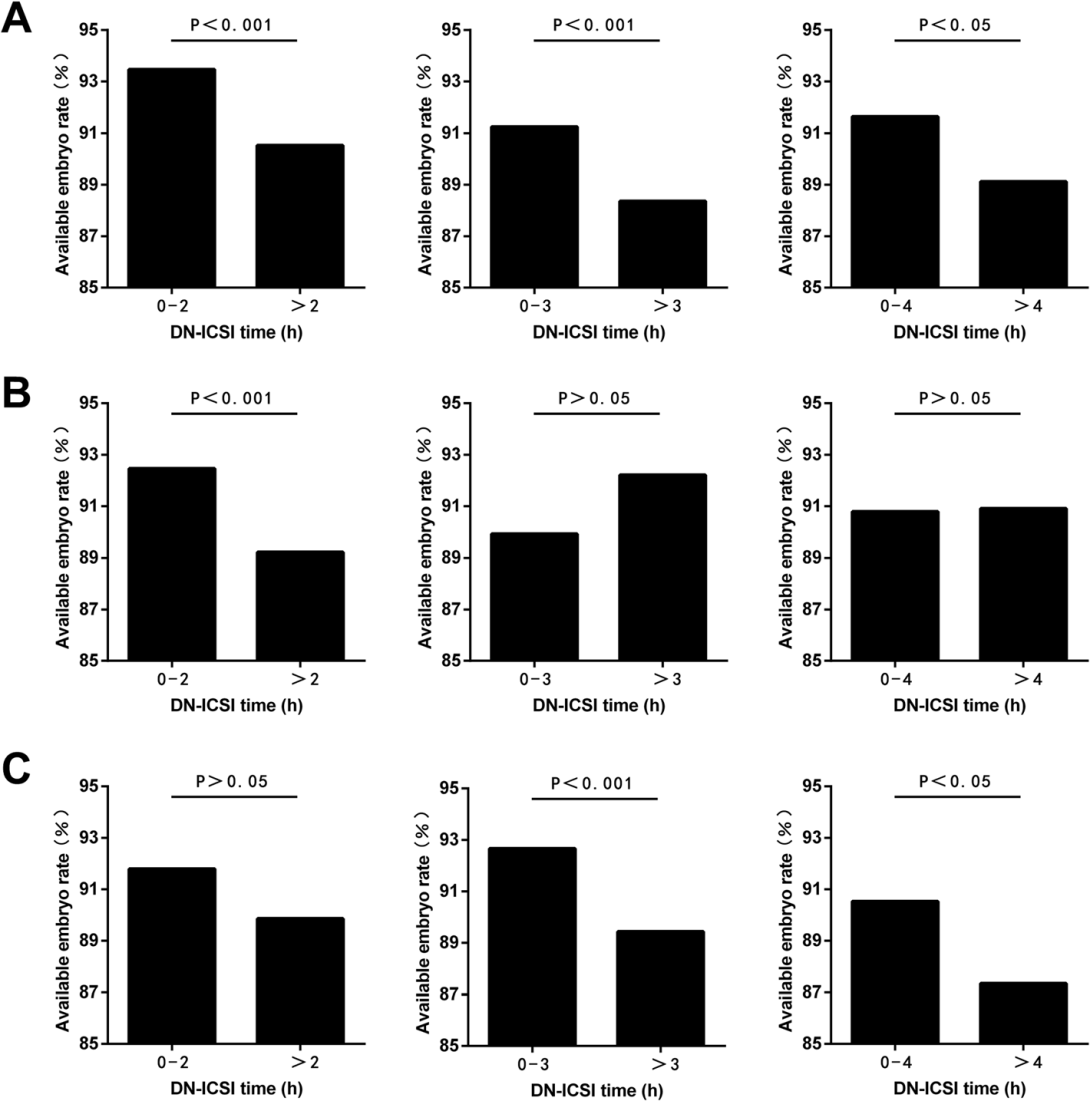
Subgroup analysis between DN-ICSI interval and available embryo rate according to HCG-OPU interval. **a** When HCG-OPU interval was < 37 h; **b** When HCG-OPU interval was 37-38 h; **c** When HCG-OPU interval was > 38 h

In order to clear up influence about oocyte aging because of different HCG trigger times on the relationship between DN-ICSI interval and reproductive outcomes, subgroup analysis according to HCG-OPU interval is exhibited in Fig. 4. All in all, the results further showed a negative correlation between prolonged DN-ICSI interval and the available embryo rate (Fig. 4).

## Discussion

Our present study discovered DN-ICSI time interval acted as an independent predictor for clinical outcomes in ICSI cycles. Indeed, most investigators have showed the relation between DN-ICSI interval and fertilization rate [6, 11, 14, 15]. Nevertheless, there are limited studies available for the influence on the clinical outcomes associated with raised DN-ICSI interval. Based on our findings, it is recommended performing ICSI within 4 h after denudation can optimize the clinical pregnancy rate in ICSI cycles.

The present study showed the increase in the fertilization rate over time of DN-ICSI interval. And the result was consistent with a recent large study [2] involving 1468 ICSI cycles which discovered the significant positive impact of the time before ICSI on fertilization. Also, Kubiak et al. [16] reported in mouse nuclear and cytoplasmic of oocytes maturation were distinguishable procedures. The process of the latter was referred as the capability of the oocyte to undergo complete activation. If performing ICSI before oocyte cytoplasm got maturation, meiosis might not resume and form of pronuclei might get a failure. Additionally, Rienzi et al. [7] showed that the optimal time for better outcomes of the fertilization rate in ICSI cycles appeared to be between 3 and 12 h after OPU, when oocytes tended to get complete cytoplasmic maturation.

Our results indicated that the increase of the fertilization rate might be caused by cytoplasmic maturation of immature oocytes and the stronger tendency of oocyte activation took about by in vitro culture, which might also result in ageing in vitro. Oocytes were usually fertilized without any delay following ovulation, and the window for appropriate fertilization time varied from species to species [1]. Normally if the oocyte did not be fertilized within the optimal interval, unfertilized oocyte could stay in the oviduct or culture medium, where it underwent a time-dependent decline in quality. And the procedure was called “oocyte aging,” which was referred as a main reason of compromised embryonic development following IVF, ICSI, and parthenogenetic activation [17, 18].

Additionally, it was found that the oocyte aging procedure was associated with changes in activity of M-phase promoting factor (MPF) [19], mitogen-activated protein kinase (MAPK) [20], concentration of calcium ions [21], and reactive oxygen species [22]. MPF was a protein kinase that played a regulatory role in oocyte meiosis and could promote progression into meiosis II [23]. Also, MPF maintained the high of activity state during the metaphase II. Besides MPF, metaphase II-arrested oocytes were exhibited by elevated levels activity of MARK which regulated the transition from meiosis I to meiosis II. In particular, both MPF and MARK were necessary in metaphase II stage arrest [24].

Carla et al. [25] observed changes of MPF and MAPK activities which showed spontaneous decrease after ovulation in-vitro culture. Kikuchi et al. [26, 27] found that along with oocytes aging, MPF converted to pre-MPF which had no activity of MPF because of the phosphorylation of cycline B and cycline-dependent kinase p34cdc2 complex. With prolonged incubation time in vitro, some oocytes were spontaneously activated which was the main manifestations of oocyte ageing due to the mode of MPF inactivation and decreasing MAPK activity [28]. In short, these changes made oocytes have ‘activation competent’. Thus, aged oocytes with less MPF might be easily activated in ICSI treatments and form pronuclei. That is the reason for an increase of fertilization rate with increasing OPU-ICSI interval [2].

Opinions about the optimal time to perform ICSI following DN varied in different researches. In 1998, van de velde et al. [10] showed that a period of time between OPU and ICSI was not related to ICSI outcomes and MII oocytes may not require further cytoplasmic maturation. Also, Jacobs et al. [14] found that the denudation and ICSI interval was not related to the ICSI outcomes. On the contrary, Boldi et al. [15] retrospectively analyzed 203 ICSI cycles, which divided into < 1 h group and 1-3 h group according to time between DN and ICSI in 2010. The data indicated that the top-quality embryo, implantation and pregnancy rates of < 1 h group were higher than 1-3 h group, while fertilization rate was not statistically different between groups. It was believed that the procedure might achieve a better outcome if ICSI was performed immediately after denudation. Besides, Catherme patrat et al. [6] performed logistic regression analysis on 110 cycles and found that in terms of the fertilization rate, an optimal time to carry out DN was within 3 h following OPU and performing ICSI soon after DN was a good choice. However, other researches did not confirm the optimal time to perform ICSI following DN.

In the present study, we chose the clinical pregnancy rate as the main outcome chosen for analysis. Interestingly, we found an opposite tendency in fertilization and clinical pregnancy rates as DN-ICSI interval increased which indicated the negative correlation of DN-ICSI interval and the clinical pregnancy rate in ICSI cycles. As previously stated, if oocytes which should have be fertilized did not be fertilized within the appropriate period, the oocytes remained within the oviduct and culture medium [29, 30]. And the oocytes would suffer aging which impaired oocyte ability. In order to find an equilibrium timing point between full cytoplasmic maturation and oocyte aging, equally, the fertilization and clinical pregnancy rate, we set different cut-off points. The implantation rate corrected for imbalance in the number of embryos transferred across groups. More importantly, the data of clinical pregnancy rate in our research further clarified the conclusions that clinical pregnancy rate was significantly higher in DN-ICSI interval < 4 h compared to DN-ICSI interval > 4 h with similar implantation rates. Especially, the interval also seemed to be reasonable for fertilization rate.

The reason for the decline in the clinical pregnancy rate over time is what we want to know. Aging after ovulation seemed to influence oocyte function including oocyte growth and recruitment, alteration, expression and storage of mature factors. These limitations influenced the developmental capacity [31]. Undoubtedly mature oocytes owned separate compartments such as the subcortical RNP domain (SCRD) and spindle chromosome complex (SCC) for storing proteins and information [31, 32]. Furthermore, RNA-binding proteins (MSY2) which was abound in the SCRD and SCC significantly reduced because of oocyte aging might disrupt the recruitment, deadenylation and degradation of developmentally important RNAs. What is more, change in the SCC-MSY2 domain and the maternal effect protein BRG1 in chromatin potentially might have an effect on spindle integrity and normal epigenetic histone modifications. In particular, the absence of H3K9 trimethylation which was an important part in histone modifications and essential for bipolar spindle formation and chromosome alignment, might result in a risk of aneuploidy [33] and the decrease of clinical pregnancy rate.

Considering that HCG administration time was the key factor for oocyte final maturation [34] which further affected denudation and ICSI timing, we also performed a subgroup analysis according to HCG-OPU interval. And the result demonstrated that when ensuring a certain number of available embryos, 4 h was still the most optimal demarcation point taking into account both HCG-OPU interval and DN-ICSI interval to obtain better clinical outcomes.

As one limitation, implantation was not considered as a primary outcome index in our study because of the different number of embryos transferred in the enrolled patients. Since implantation potential was a more accurate indicator for oocyte vitality, in our future work, we will aim to explore a correlation between DN-ICSI internal and embryo implantation chance in a well-designed single-embryo-transfer cohort study.

Compared with other available literatures, our study should be more convinced. As we know, distinct oocyte or sperm genetic disease greatly affect results, we set exclusion criteria to carry on an accurate analyze and avoid bias for the study. What’s more, another strength is that our research includes the selection of a great number of infertile patients analysed, valid comparisons of outcomes in the cycles of different groups and detailed times from HCG to ICSI recorded by the exact operator independently. In addition, we also adopt measures of subgroup analysis to proclaim the relationship between DN-ICSI and available embryo rates in terms of an interval between HCG trigger and oocyte retrieval.

## Conclusions

In conclusion, this study indicates that DN-ICSI interval significantly affects the clinical outcomes and the optimal timing for ICSI is within 4 h after oocyte denudation for excellent laboratory and reproductive results in ICSI cycles. DN-ICSI time interval can act as an independent predictor for clinical outcomes in ICSI cycles.

## Data Availability

All data from the current study supporting the conclusions are presented in the article.

## Abbreviations

COC: Cumulus cell-oocyte complex
COH: Controlled ovarian hyperstimulation
DN: Denudation
FSH: Follicle-stimulating hormone
HCG: Human chorionic gonadotropin
ICSI: Intracytoplasmic sperm injection
MPF: M-phase promoting factor
MAPK: Mitogen-activated protein kinase
OPU: Oocyte pick up
